# Zhaoshumycins A and B, Two Unprecedented Antimycin-Type Depsipeptides Produced by the Marine-Derived *Streptomyces* sp. ITBB-ZKa6

**DOI:** 10.3390/md19110624

**Published:** 2021-11-05

**Authors:** Zhikai Guo, Shiying Ma, Salman Khan, Hongjie Zhu, Bo Zhang, Shiqing Zhang, Ruihua Jiao

**Affiliations:** 1 Institute of Tropical Bioscience and Biotechnology, Chinese Academy of Tropical Agricultural Sciences, Haikou 571101, China; zhangshiqing@itbb.org.cn; 2 Hainan Key Laboratory of Conservation and Utilization of Tropical Agricultural Bioresources, Hainan Institute of Tropical Agricultural Resources, Chinese Academy of Tropical Agricultural Sciences, Haikou 571101, China; 3 State Key Laboratory of Pharmaceutical Biotechnology, Institute of Functional Biomolecules, School of Life Sciences, Nanjing University, Nanjing 210023, China; MG1630053@smail.nju.edu.cn (S.M.); Sallo.khan3445@gmail.com (S.K.); hongjie6@163.com (H.Z.); bzhang@nju.edu.cn (B.Z.)

**Keywords:** antimycin, depsipeptide, cytotoxic activity, *Streptomyces* sp., marine actinomycetes

## Abstract

Marine actinomycetes are prolific chemical sources of complex and novel natural products, providing an excellent chance for new drug discovery. The chemical investigation of the marine-derived *Streptomyces* sp. ITBB-ZKa6, from Zhaoshu island, Hainan, led to the discovery of two unique antimycin-type depsipeptides, zhaoshumycins A (**1**) and B (**2**), along with the isolation of the four known neoantimycins A (**3**), F (**4**), D (**5**), and E (**6**). The structures of the new compounds **1** and **2** were elucidated on the basis of the analysis of diverse spectroscopic data and biogenetic consideration. Zhaoshumycins A (**1**) and B (**2**) represent a new class of depsipeptides, featuring two neoantimycin monomers (only neoantimycin D or neoantimycins D and E) linked to a 1,4-disubstituted benzene ring via an imino group. Initial toxicity tests of **1**–**6** in MCF7 human breast cancer cells revealed that compounds **5** and **6** possess weak cytotoxic activity. Further structure–activity relationship analysis suggested the importance of the NH_2_ group at C-34 in **5** and **6** for cytotoxicity in MCF7 cells.

## 1. Introduction

Marine-derived actinomycetes are an excellent treasure house of structurally novel and biologically active natural products with potent medicinal properties [1,2,3,4,5]. In recent years, a various complex secondary metabolites, exemplified by the recently reported atratumycin, lobophorin, angucycline glycosides, tetrahydroisoquinolines, and polycyclic tetramate macrolactams, were isolated from marine-derived *Streptomyces* strains residing on or in mollusks, algae, corals, and sediments, indicating the marine-derived genus *Streptomyces* as a predominant source of chemically diverse and bioactive natural products [6,7,8,9,10]. Antimycins are a family of depsipeptide compounds typically discovered in actinomycetes, displaying a broad range of promising biological activities including anticancer, antiviral, antifungal, and nematocidal effects [11]. They are generated by a hybrid multimodular protein complex of non-ribosomal peptide synthetase and polyketide synthase assembly lines and have a common core structure composed of a macrocyclic ring with an amide linkage to a 3-formamidosalicylate unit [12,13,14]. This kind of depsipeptide natural products have attracted researchers’ attention due to their diverse structures and important bioactivities. Structurally, members of the antimycin family differ by the varying size of their macrocyclic ring and modifications of their ester forming α-hydroxy acid components. So far, four major antimycin-type depsipeptides that differ by ring size, including 9-, 12-, 15-, and 18-membered macrocyclic ring-bearing antimycins, have been reported.

In our continuous work to exploit structurally novel and bioactive natural products from actinomycetes living in underexplored ecological niches [15,16,17,18], a marine-derived actinomycete strain identified as *Streptomyces* sp. ITBB-ZKa6 was investigated. Examining the fermentation extract of this strain, we discovered the new antimycin-type depsipeptides **1** and **2**. Herein, we report the isolation, structure elucidation, and cytotoxic activities of zhaoshumycins A (**1**) and B (**2**) from this strain.

## 2. Results

**Figure 1:**
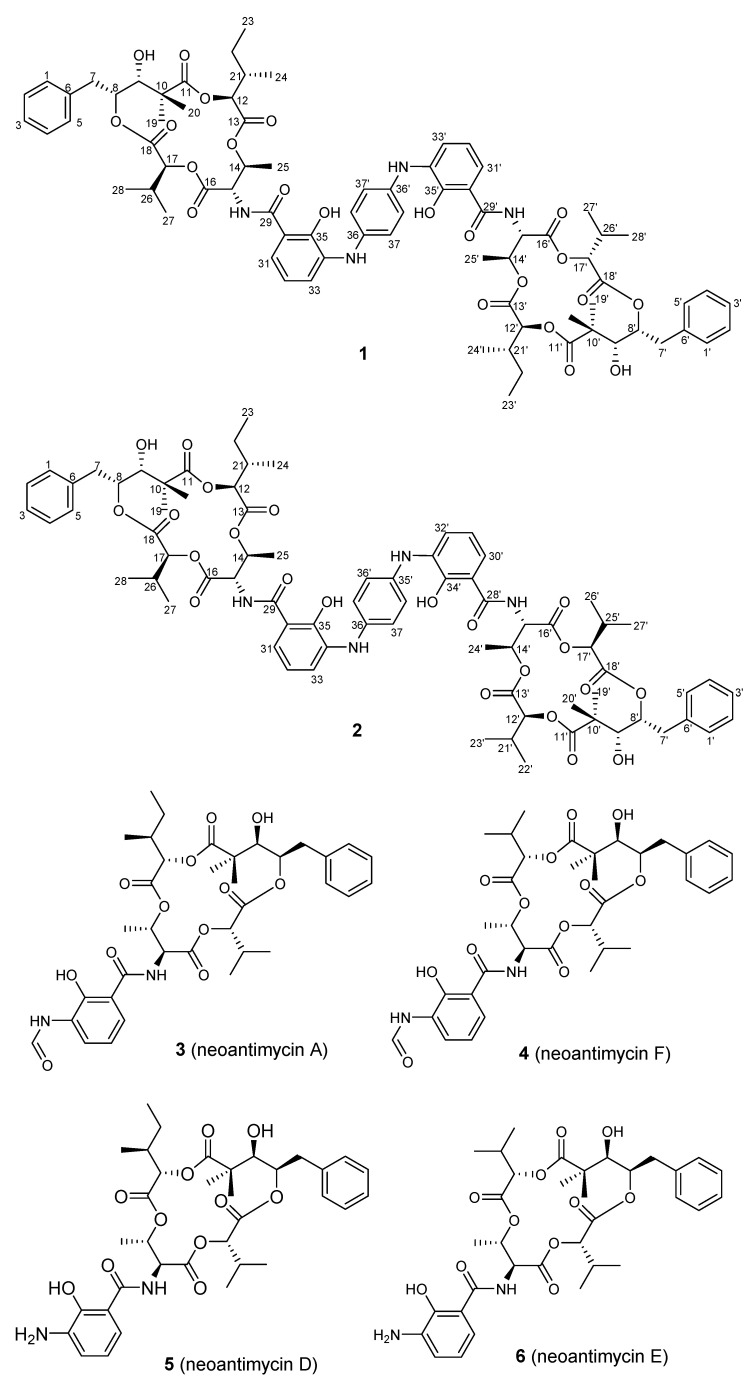
Chemical structures of the isolated compounds **1–6**.

The producing strain ITBB-ZKa6 was isolated from a marine sample obtained in Zhaoshu island, Sansha, Hainan at a depth of 1 m. We performed 16S rRNA sequence analysis that showed it to be similar to *Streptomyces* species. HPLC analysis of the fermentation extracts of this strain revealed an abundant metabolite profile in medium F, showing a series of compounds with complex UV/VIS absorptions. Subsequent large-scale fermentation of *Streptomyces* sp. ITBB-ZKa6 in medium F, followed by extraction of the cultures with EtOAc, chromatographic purification including silica gel, ODS, Sephadex LH20 chromatography, and semi-preparative HPLC, resulted in the discovery of compounds **1**–**6** (Figure 1). The known neoantimycin A (**3**), neoantimycin F (**4**), neoantimycin D (**5**). and neoantimycin E (**6**) were characterized by comparing their MS and NMR spectroscopic data with those reported (Figures S16–S19, Tables S1–S4) [12,13,19].

Compound **1** was isolated as a yellow amorphous powder. High-resolution electrospray ionization mass spectrometry analysis (HRESIMS) of **1** gave an [M + Na]^+^ ion at *m/z* 1437.6252, which corresponds to a calculated molecular formula of C_76_H_94_N_4_O_22_ (calcd for C_76_H_94_N_4_O_22_Na, 1437.6252) (Figure S24), requiring 32 degrees of unsaturation. The ^1^H (Figure S1), ^13^C (Figure S2), and DEPT135 (Figure S3) NMR data (Table 1) in combination with HSQC (Figure S5) identified 10 aromatic methine signals (*δ*_C_/*δ*_H_: 129.2/7.21, 129.2/7.21, 128.7/7.27, 128.7/7.27, 126.9/7.21, 121.8/7.15, 121.8/7.15, 114.7/7.02, 118.8/6.81, and 116.2/7.30), six non-protonated carbons (*δ*_C_: 150.2, 136.8, 136.6, 135.0, 112.7, and 45.4), and five carbonyl carbons (*δ*_C_: 176.9, 170.8, 168.5, 168.4, and 168.2). Further interpretation of the NMR data revealed the presence of eight methine groups (*δ*_C_/*δ*_H_: 79.1/3.20, 76.6/5.45, 75.2/4.67, 72.6/5.74, 71.8/5.52, 55.1/5.16, 36.0/1.96, 30.7/1.80), two aliphatic methylene groups (*δ*_C_/*δ*_H_: 40.3/3.16, 2.94 and 24.8/1.52, 1.20), and seven methyl groups (*δ*_C_/*δ*_H_: 26.9/1.40, 21.9/1.30, 18.7/0.82, 16.3/1.35, 16.1/0.46, 14.3/0.89, 10.9/0.88). Interestingly, the ^13^C NMR data accounted for only half of the carbons observed in the molecular formula of **1**, indicating its symmetry in the structure. The ^1^H and ^13^C NMR data of **1** were almost identical to those of neoantimycin D (**5**), except for the presence of two downfield-shifted carbon resonances of C-33 and C-34 in the 1,2,3-trisubstituted benzene ring of **1,** and extra NMR data attributed them to a 1,4-disubstituted benzene ring.

**Figure 2:**
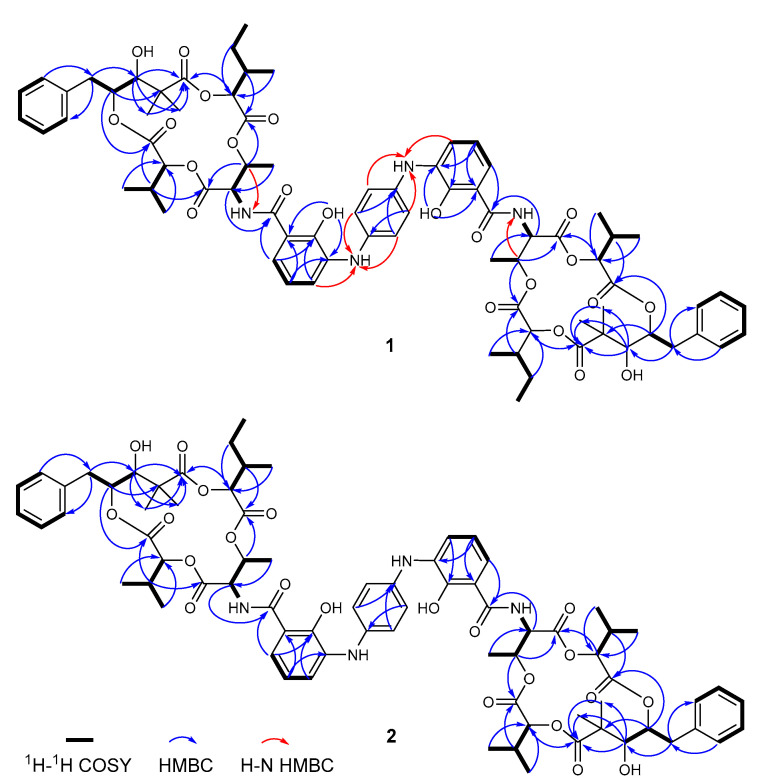
Key 2D NMR correlations for zhaoshumycins A (**1**) and B (**2**).

The presence of a monosubstituted benzene ring was disclosed by the ^1^H-^1^H COSY (Figure S4) correlations from H-2 (*δ*_H_ 7.27) to H-1 (*δ*_H_ 7.21) and H-3 (*δ*_H_ 7.21) and from H-4 (*δ*_H_ 7.27) to H-3 and H-5 (*δ*_H_ 7.21) (Figure 2). One spin system from H_2_-7 (*δ*_H_ 3.16 and 2.94) to H-9 (*δ*_H_ 3.20) was identified from the ^1^H-^1^H COSY spectrum. HMBC correlations (Figure S6) from H-8 (*δ*_H_ 5.52) to C-10 (*δ*_C_ 45.4) and from H-9 to the carbonyl C-11 (*δ*_C_ 176.9) enabled the assignment of fragment C-7 (*δ*_C_ 40.3)/C-8 (*δ*_C_ 71.8)/C-9 (*δ*_C_ 79.1)/C-10/C-11, where two methyl groups were positioned at C-10 by the observation of HMBC correlation signals from H_3_-19 (*δ*_H_ 1.30) and H_3_-20 (*δ*_H_ 1.40) to C-11 and from H-9 to C-19 (*δ*_C_ 21.9) and C-20 (*δ*_C_ 26.9). The key HMBC correlations from H-1 and H-5 to C-7 and from H_2_-7 to C-1 and C-5 (*δ*_C_ 129.2) indicated the linkage of this fragment with the 1,2,3-trisubstituted benzene ring via the connectivity of C-6 (*δ*_C_ 136.8)/C-7. The COSY correlations from H-12 (*δ*_H_ 4.67) to H-21 (*δ*_H_ 1.96), from H-21 to H_2_-22 (*δ*_H_ 1.52 and 1.20) and H_3_-24 (*δ*_H_ 0.89), and from H-22 to H_3_-23 (*δ*_H_ 0.88), and the key HMBC correlations from H_2_-22 and H_3_-24 to C-12 (*δ*_C_ 75.2) and from H-21 to the carbonyl C-13 (*δ*_C_ 168.2) confirmed the presence of a 2-hydroxy-3-methyl-pentanoic acid moiety. The connectivity of C-25 (*δ*_C_ 16.3)/C-14 (*δ*_C_ 72.6)/C-15 (*δ*_C_ 55.1)/C-16 (*δ*_C_ 168.4) was established by the COSY correlations from H-14 (*δ*_H_ 5.74) to H-15 (*δ*_H_ 5.16) and H-25 (*δ*_H_ 1.35) and the HMBC correlation from H-14 to the carbonyl C-16. A fragment of 2-hydroxy-3-methyl-butanoic acid could also be deduced by the COSY correlations of H-17 (*δ*_H_ 5.45) with H-26 (*δ*_H_ 1.80) and of H-26 with H_3_-27 (*δ*_H_ 0.46) and H_3_-28 (*δ*_H_ 0.82) and HMBC correlations from H_3_-27 and H_3_-28 to C-17 (*δ*_C_ 76.6) and form H-26 to the carbonyl C-18 (*δ*_C_ 168.5).

The characteristic ^3^*J* HMBC correlations from H-8 to C-18, H-12 to C-11, H-14 to C-13, and H-26 to C-16 allowed the construction of a 15-membered macrocyclic ester ring. In addition, the ^1^H-^1^H COSY correlations from H-32 (*δ*_H_ 6.81) to H-31 (*δ*_H_ 7.02) and H-33 (*δ*_H_ 7.30) and the HMBC correlations from H-32 to C-30 (*δ*_C_ 112.7) and C-34 (*δ*_C_ 135.0) and from H-31 and C-33 (*δ*_C_ 116.2) to C-35 (*δ*_C_ 150.2) elucidated a 1,2,3-trisubstituted benzene ring. The attachment of the carbonyl C-29 (*δ*_C_ 170.8) and the chelated hydroxy group (*δ*_H_ 12.50) to the C-30 (*δ*_C_ 112.7) and C-35 of the trisubstituted benzene ring, respectively, was evidenced by the HMBC correlations of H-31 to C-29 and of 35-OH to C-30 and C-34. The key HMBC correlation of H-15 with C-29 and the H–N HMBC correlation (Figure S8) of H-14 with nitrogen (15-N, *δ_N_* 100.7) allowed the connectivity of the 15-membered macrocyclic ester ring with the trisubstituted benzene ring through amide linkage, which is identical to the core structure of neoantimycin D (**5**). In the H-N HMBC spectrum, H-33 and H-37 (*δ*_H_ 7.15) were observed to correlate with the same nitrogen (*δ_N_* 76.6), suggesting that the trisubstituted benzene ring was linked to the 1,4-disubstituted benzene ring by an imino group. Since compound **1** is a dimer, two neoantimycin D monomers appeared connected with the 1,4-disubstituted benzene ring through imino groups at C-34 and C-34′. Thus, the complete planar structure of **1** was established, as shown in Figure 1; the compound was named zhaoshumycin A.

Compound **2** was obtained as a yellow amorphous powder. The molecular formula of **2** was determined to be C_75_H_92_N_4_O_22_, as deduced by its HRESIMS analysis (observed an [M + Na]^+^ ion peak at *m/z* 1423.6092, calcd 1423.6095) (Figure S25). The NMR and UV spectra were very similar to those of **1**, indicating they share a similar core structure. Comparing the ^1^H (Figure S9), ^13^C (Figure S10), and DEPT135 (Figure S11) NMR data of **2** (Table 2) with those of **1** made it clear that they are almost identical except for the presence of an upfield-shifted methine signal at *δ*_H_: 4.58 (d, 7.9) (H-12′) and two downfield-shifted methyl proton signals at *δ*_H_: 0.92 (d, 6.8) (H_3_-22′) and 0.97 (d, 6.8) (H_3_-23′) in **2**. Detailed analysis of the ^1^H-^1^H COSY spectrum revealed these two methyl groups (H_3_-22′ and H_3_-23′) displayed correlations with H-12′. This result suggested the presence of 2-hydroxy-3-methyl-butanoic acid instead of 2-hydroxy-3-methyl-pentanoic acid in **2**. Comprehensive analysis of the ^1^H, ^13^C, HSQC (Figure S13), HMBC (Figure S14), and ^1^H-^1^H COSY (Figure S12) spectra of **2** (Figure 2) allowed its planar structure to be determined as shown; the compound was named zhaoshumycin B.

The relative stereochemistry of **1** and **2** was established by NOESY (Figure S7 and Figure S15) NMR spectrum analysis, which implied that **1** and **2** have the same configurations as those reported for neoantimycin A–F [12,13]. The optical rotation of **1** (+100, *c* 0.1, MeOH) and **2** (+90, *c* 0.1, MeOH) were also similar to those of neoantimycin D (**5**) and E (**6**) [12], which were co-isolated from the extract. Taking this into consideration, the absolute structures of **1** and **2** were assigned, as shown in Figure 1, on the basis of a common biosynthetic origin shared with **5** and **6**.

Antimycin-type natural products have been reported to possess promising anticancer activities against a panel of human cancer cell lines [14]. To obtain preliminary information of the cytotoxic activity of compounds **1**–**6**, we assessed their toxicity toward the MCF7 human breast cancer cell line. Only compounds **5** and **6** showed activity at a concentration of 50 µM, with an inhibition rate of 91.08% and 90.53%, respectively. However, no obvious activity was observed for compounds **1**–**4** under our experimental conditions. To further understand the structure–activity relationship, we speculated that the loss of toxicity for compounds **1**–**4** in MCF7 cells could be potentially attributed to the derivatization of the NH_2_ group at C-34, clearly signifying its importance in biological activity.

## 3. Materials and Methods

### 3.1. General Experimental Procedures

Column chromatography (CC) was carried out with silica gel (200–300 mesh, Qingdao Marine Chemical Inc., Qingdao, China), ODS (40–70 µm, Merck Company, Darmstadt, Germany), and Sephadex LH-20 (GE Healthcare Bio-Sciences AB, Uppsala, Sweden). Thin-layer chromatography (TLC) was performed on precoated glass plates (silica gel GF_254_, 10–20 µm, Qingdao Marine Chemical Inc., Qingdao, China). Semi-preparative HPLC was performed on a Hitachi HPLC system equipped with a L-7100 pump, a L-7400 UV detector, and an ODS-2 Hypersil column (5 µm, 250 × 10.0 mm, Thermo Fisher Scientific, Waltham, MA, USA). High-resolution mass spectra (HRESIMS) were acquired on an Agilent 6210 TOF LC-MS instrument (Agilent Technologies Inc., Palo Alto, CA, USA). Optical rotations were measured in methanol using a Rudolph Autopol IV automatic polarimeter (Rudolph Research Analytical, Hackettstown, NJ, USA). UV spectra were recorded on a Nanodrop2000 spectrometer (Thermo Scientific, Wilmington, DE, USA). One- and two-dimensional (1D and 2D) NMR spectra were collected on a Bruker DPX-400 NMR spectrometer (400 MHz for ^1^H NMR and 100 MHz for ^13^C NMR) and a Bruker DRX-600 spectrometer (600 MHz for ^1^H NMR and 150 MHz for ^13^C NMR) (Bruker Corporation, Karlsruhe, Germany). The chemical shifts of ^1^H and ^13^C NMR spectra are given in *δ* (ppm) and referenced to the solvent signal (CDCl_3_, *δ*_H_ 7.26 and *δ*_C_ 77.16). Coupling constants (J) are reported in Hz.

### 3.2. Strain Isolation and Identification

The actinomycete strain ITBB-ZKa6 was isolated by Z.G. from a marine sediment sample near a marine invertebrate, a yellow sponge that lived 1 m underwater, collected in Zhaoshu island, Sansha, Hainan, P.R. China. It was identified as a member of the *Streptomyces* genus by its morphological characteristics and 16S rRNA sequence analysis and comparison with other reported sequences in the NCBI GenBank database. The 16S rRNA sequence of *Streptomyces* sp. ITBB-ZKa6 was deposited in GenBank (accession no. OK381602), and this strain is preserved in the Institute of Tropical Bioscience and Biotechnology, Chinese Academy of Tropical Agricultural Sciences.

### 3.3. Extraction and Isolation

The strain *Streptomyces* sp. ITBB-ZKa6 was cultivated in TSB liquid medium containing sea salt (consisting of 15 g/L tryptone, 5 g/L soy protein, 5 g/L NaCl, 27 g/L sea salt and 1 L sterilized deionized water) in 250 mL Erlenmeyer flasks for 2 days on a rotary shaker at 140 rpm/min at 28 °C. Then, 50 mL of the cultured medium was inoculated into F medium containing sea salt (consisting of sucrose 20 g, glucose 10 g, casamino acids 0.1 g, yeast extract 5 g, MOPS 5 g, K_2_SO4 0.25 g, MgCl_2_·6H_2_O 1 g, trace element(10×) 100 μL, 27 g sea salt, in 1 L sterilized deionized water) in a 1 L Erlenmeyer flask and cultivated at 28 °C for 10 days on a rotary shaker at 140 rpm/min. The whole culture of ITBB-ZKa6 was then extracted with ethyl acetate (EtOAc) for three times. The EtOAc extract was dried in vacuo to yield 10 g of crude extract. The entire crude extract was subjected to silica gel CC using gradient elution with petroleum ether/EtOAc mixtures (*v/v*, 100:4, 100:8, 100:16, 100:32, 1:1, 0:100) and EtOAc/MeOH mixtures (*v/v*, 100:4, 100:8, 100:16, 100:32, 1:1, 0:100) to obtain 15 fractions (Fr.1–Fr.15). Fr.5 and Fr.7 were subsequently subjected to ODS CC, Sephadex LH20 CC, and purified by semi-preparative reverse-phase HPLC using a gradient solvent system from 40% to 80% MeOH in H_2_O (2 mL/min, UV *λ*_max_ 254 nm) over 45 min to afford compounds **1** (4 mg), **2** (2 mg), **3** (9 mg), **4** (6 mg), **5** (10 mg), and **6** (6 mg).

Zhaoshumycin A (**1**): Yellow amorphous powder; [*α*]_D_^25^ + 100 (*c* 0.1, MeOH); UV (MeOH) *λ*_max_ (log *ε*) 217 (5.72), 242 (5.75), 363 (5.21), 467 (4.69) nm; ^1^H and ^13^C NMR spectroscopic data, see Table 1; HR-ESI-MS *m/z* 1437.6252 [M + Na]^+^ (calcd for C_76_H_94_N_4_O_22_Na, 1437.6252).

Zhaoshumycin B (**2**): Yellow amorphous powder; [*α*]_D_^25^ + 90 (*c* 0.1, MeOH); UV (MeOH) *λ*_max_ (log *ε*) 214 (5.56), 242 (5.54), 291 (5.27), 340 (4.95), 365 (5.00) nm; ^1^H and ^13^C NMR spectroscopic data, see Table 2; HR-ESI-MS *m/z* 1423.6089 [M + Na]^+^ (calcd for C_75_H_92_N_4_O_22_Na, 1423.6095).

### 3.4. Cytotoxic Activity Test

The cytotoxic activity of compounds **1**–**6** was in vitro evaluated using the human breast cancer cell line MCF7 (purchased from the Jiangsu Provincial Center for Disease Prevention and Control) according to the previously reported MTT method [20]. Dimethyl sulfoxide (DMSO) was used to dissolve the tested compounds.

## 4. Conclusions

During the analysis of novel natural products from the fermentation extract of actinomycetes from underexplored ecological niches, we discovered two unique dimeric antimycin-type depsipeptides, zhaoshumycins A (**1**) and B (**2**), together with the four known neoantimycins A (**3**), F (**4**), D (**5**), and E (**6**). These compounds were obtained from the marine-derived *Streptomyces* sp. ITBB-ZKa6 from Zhaoshu island, Hainan. Zhaoshumycins A (**1**) and B (**2**) were identified as a new class of depsipeptides, featuring two neoantimycin moieties (only neoantimycin D or neoantimycins D and E) linked to a 1,4-disubstituted benzene ring via two imino groups. To the best of our knowledge, Zhaoshumycins A (**1**) and B (**2**) are the first examples of dimeric antimycin-type depsipeptides found in nature, representing a new class of this kind of natural products. This new structural feature expands the structural diversity of antimycin-type depsipeptides. A preliminary in vitro cytotoxicity test of **1**–**6** in MCF7 human breast cancer cells revealed that only compounds **5** and **6** possess a weak cytotoxic activity, indicating the NH_2_ group at C-34 in **5** and **6** could be essential for cytotoxic activity in MCF7 cells. The discovery of **1** and **2** highlights the potential of marine actinomycetes as a prolific source of novel natural products for drug discovery.

## Figures and Tables

**Table 1:** ^1^H (600 MHz) and ^13^C (150 MHz) NMR data for compound **1** in CDCl_3_^a^.

Position	*δ*_C_, Type	*δ*_H_, Mult. (*J* in Hz)	Position	*δ*_C_, Type	*δ*_H_, Mult. (*J* in Hz)
1, 1′	129.2, CH	7.21, d (6.5)	21, 21′	36.0, CH	1.96, m
2, 2′	128.7, CH	7.27, t (6.5)	22, 22′	24.8, CH_2_	1.52, m; 1.20, m
3, 3′	126.9, CH	7.21, t (6.5)	23, 23′	10.6, CH_3_	0.88, overlap
4, 4′	128.7, CH	7.27, t (6.5)	24, 24′	14.3, CH_3_	0.89, overlap
5, 5′	129.2, CH	7.21, d (6.5)	25, 25′	16.3, CH_3_	1.35, d (6.5)
6, 6′	136.8, C		26, 26′	30.7, CH	1.80, m
7, 7′	40.3, CH_2_	2.94, dd (14.0, 9.6); 3.16, dd (14.0, 9.6)	27, 27′	16.1, CH_3_	0.46, d (6.9)
8, 8′	71.8, CH	5.52, dd (9.6, 5.8)	28, 28′	18.7, CH_3_	0.82, d (6.9)
9, 9′	79.1, CH	3.20, s	29, 29′	170.8, C	
10, 10′	45.4, C		30, 30′	112.7, C	
11, 11′	176.9, C		31, 31′	114.7, CH	7.02, d (7.8)
12, 12′	75.2, CH	4.67, d (8.3)	32, 32′	118.8, CH	6.81, t (7.8)
13, 13′	168.2, C		33, 33′	116.2, CH	7.30, d (7.8)
14, 14′	72.6, CH	5.74, dd (6.5, 2.6)	34, 34′	135.0, C	
15, 15′	55.1, CH	5.16, dd (8.9, 2.6)	35, 35′	150.2, C	
16, 16′	168.4, C		35-OH, 35′-OH		12.50, brs
17, 17′	76.6, CH	5.45, d (3.5)			
18, 18′	168.5, C		36, 36′	136.6, C	
19, 19′	21.9, CH_3_	1.30, s	37, 37′	121.8, CH	7.15, overlap
20, 20′	26.9, CH_3_	1.40, s	38, 38′	121.8, CH	7.15, overlap

^a^ Assignments based on 2D NMR experiments.

**Table 2:** ^1^H (400 MHz) and ^13^C (100 MHz) NMR data for compound **2** in CDCl_3_^a^.

Position	*δ*_C_, Type	*δ*_H_, Mult. (*J* in Hz)	Position	*δ*_C_, Type	*δ*_H_, Mult. (*J* in Hz)
1	129.2, CH	7.21, d (6.5)	1′	129.2, CH	7.21, d (6.5)
2	128.7, CH	7.27, m	2′	128.7, CH	7.27, m
3	126.9, CH	7.21, t (6.5)	3′	126.9, CH	7.21, t (6.5)
4	128.7, CH	7.27, m	4′	128.7, CH	7.27, m
5	129.2, CH	7.21, d (6.5)	5′	129.2, CH	7.21, d (6.5)
6	136.8, C		6′	136.8, C	
7	40.3, CH_2_	2.94, dd (14.0,5.7); 3.16, dd (14.0,9.6)	7′	40.3, CH_2_	2.94, dd (14.0, 5.7); 3.16, dd (14.0, 9.6)
8	71.8, CH	5.52, dd (9.6,5.8)	8′	71.8, CH	5.52, dd (9.6, 5.8)
9	79.1, CH	3.20, s	9′	79.1, CH	3.20, s
10	45.4, C		10′	45.4, C	
11	176.9, C		11′	176.9, C	
12	75.2, CH	4.67, d (8.3)	12′	75.2, CH	4.58, d (7.9)
13	168.2, C		13′	168.2, C	
14	72.6, CH	5.74, dd (6.5,2.6)	14′	72.6, CH	5.74, dd (6.5, 2.6)
15	55.1, CH	5.15, dd (8.9,2.6)	15′	55.1, CH	5.15, dd (8.9, 2.6)
16	168.4, C		16′	168.4, C	
17	76.6, CH	5.45, d (3.5)	17′	76.6, CH	5.45, d (3.5)
18	168.5, C		18′	168.5, C	
19	21.9, CH_3_	1.30, s	19′	21.9, CH_3_	1.30, s
20	26.9, CH_3_	1.41, d (2.7)	20′	26.9, CH_3_	1.41, d (2.7)
21	36.0, CH	1.97, m	21′	36.0, CH	1.97, m
22	24.8, CH_2_	1.51, m; 1.20, m	22′	10.6, CH_3_	0.92, d (6.8)
23	10.6, CH_3_	0.88, m	23′	14.3, CH_3_	0.97, d (6.8)
24	14.3, CH_3_	0.89, m	24′	16.3, CH_3_	1.35, d (6.5)
25	16.3, CH_3_	1.35, d (6.5)	25′	30.7, CH	1.81, m
26	30.7, CH	1.81, m	26′	16.1, CH_3_	0.46, d (6.8)
27	16.1, CH_3_	0.46, d (6.8)	27′	18.7, CH_3_	0.82, d (6.8)
28	18.7, CH_3_	0.82, d (6.8)	28′	170.8, C	
29	170.8, C		29′	112.7, C	
30	112.7, C		30′	114.7, CH	7.01, m
31	114.7, CH	7.01, m	31′	118.8, CH	6.81, t (7.8)
32	118.8, CH	6.81, t (7.8)	32′	116.2, CH	7.30, d (7.8)
33	116.2, CH	7.30, d (7.8)	33′	135.0, C	
34	135.0, C		34′	150.2, C	
35	150.2, C		34′-OH		12.50, brs
35-OH		12.50, brs	35′	136.6, C	
36	136.6, C		36′	121.8, CH	7.15, s
37	121.8, CH	7.15, s	37′	121.8, CH	7.15, s
38	121.8, CH	7.15, s			

^a^ Assignments based on 2D NMR experiments.

## Data Availability

The authors declare that all data of this study are available within the article and its Supplementary Materials file or from the corresponding authors upon request.

## Supplementary Materials

The following are available online at https://www.mdpi.com/article/10.3390/md19110624/s1, 1D and 2D NMR spectra (Figure S1–Figure S15) and MS spectra (Figure S24, Figure S25) for compounds **1**–**2**; NMR data (Table S1–Table S4), 1H NMR spectra (Figure S16–Figure S19) and structures (Figure S20–Figure S23) for compounds **3**–**6**.
