# Supplementary material for: Zhaoshumycins A and B, Two Unprecedented Antimycin-Type Depsipeptides Produced by the Marine-Derived Streptomyces sp. ITBB-ZKa6
Source: Mar Drugs. 2021 Nov 5;19(11):624. doi: 10.3390/md19110624 (PMC8623215; doi:10.3390/md19110624)

Supporting Information

Zhaoshumycins A and B, Two Unprecedented Antimycin-Type Depsipeptides Produced by a Marine-Derived *Streptomyces* sp. ITBB-ZKa6

Zhikai Guo [1,2,†,*], Shiying Ma [3,†], Salman Khan [3], Hongjie Zhu [3], Bo Zhang [3], Shiqing Zhang [1] and Ruihua Jiao [3,*]

[1] Institute of Tropical Bioscience and Biotechnology, Chinese Academy of Tropical Agricultural Sciences, Haikou 571101, China; zhangshiqing@itbb.org.cn (S.Z.)

[2] Hainan Key Laboratory of Conservation and Utilization of Tropical Agricultural Bioresources, Hainan Institute of Tropical Agricultural Resources, Chinese Academy of Tropical Agricultural Sciences, Haikou 571101, China

[3] State Key Laboratory of Pharmaceutical Biotechnology, Institute of Functional Biomolecules, School of Life Sciences, Nanjing University, Nanjing 210023, China; MG1630053@smail.nju.edu.cn (S.M.); Sallo.khan3445@gmail.com (S.K.); hongjie6@163.com (H.Z.); bzhang@nju.edu.cn (B.Z.)

* Correspondence: guozhikai@itbb.org.cn (Z.G.); rhjiao@nju.edu.cn (R.J.)

† These authors contributed equally to this work.

Figure S1. ^{1}H NMR spectrum of **1** in $CDCl_3$ (600 MHz)

Figure S2. ^{13}C NMR spectrum of **1** in $CDCl_3$ (150 MHz)

Figure S3. DEPT135 NMR spectrum of **1** in $CDCl_3$ (600 MHz)

Figure S4. ^{1}H-^{1}H COSY NMR spectrum of **1** in $CDCl_3$ (600 MHz)

Figure S5. HSQC NMR spectrum of **1** in $CDCl_3$ (600 MHz)

Figure S6. HMBC NMR spectrum of **1** in $CDCl_3$ (600 MHz)

Figure S7. NOESY NMR spectrum of **1** in $CDCl_3$ (600 MHz)

Figure S8. H-N HMBC NMR spectrum of **1** in $CDCl_3$ (600 MHz)

Figure S9. ^{1}H NMR NMR spectrum of **2** in $CDCl_3$ (400 MHz)

Figure S10. ^{13}C NMR spectrum of **2** in $CDCl_3$ (100 MHz)

Figure S11. DEPT135 NMR spectrum of **2** in $CDCl_3$ (400 MHz)

Figure S12. ^{1}H-^{1}H COSY NMR spectrum of **2** in $CDCl_3$ (400 MHz)

Figure S13. HSQC NMR spectrum of **2** in $CDCl_3$ (400 MHz)

Figure S14. HMBC NMR spectrum of **2** in $CDCl_3$ (400 MHz)

Figure S15. NOESY NMR spectrum of **2** in $CDCl_3$ (400 MHz)

Figure S16. ^{1}H NMR spectrum of **3** in $CDCl_3$ (400 MHz)

Figure S17. ^{1}H NMR spectrum of **4** in $CDCl_3$ (400 MHz)

Figure S18. ^{1}H NMR spectrum of **5** in $CDCl_3$ (400 MHz)

Figure S19. ^{1}H NMR spectrum of **6** in $CDCl_3$ (400 MHz)

Figure S20. The structure of compound **3**

Table S1. NMR data for compound **3** in $CDCl_3$ (400 MHz)

Figure S21. The structure of compound **4**

Table S2. ^{1}H NMR data for compound **4** in $CDCl_3$ (400 MHz)

Figure S22. The structure of compound **5**

Table S3. NMR data for compound **5** in $CDCl_3$ (400 MHz)

Figure S23. The structure of compound **6**

Table S4. ^{1}H NMR data for compound **6** in $CDCl_3$ (400 MHz)

Figure S24. MS spectrum of **1**

Figure S25. MS spectrum of **2**

Figure S1. ^{1}H NMR spectrum of **1** in $CDCl_3$ (600 MHz)

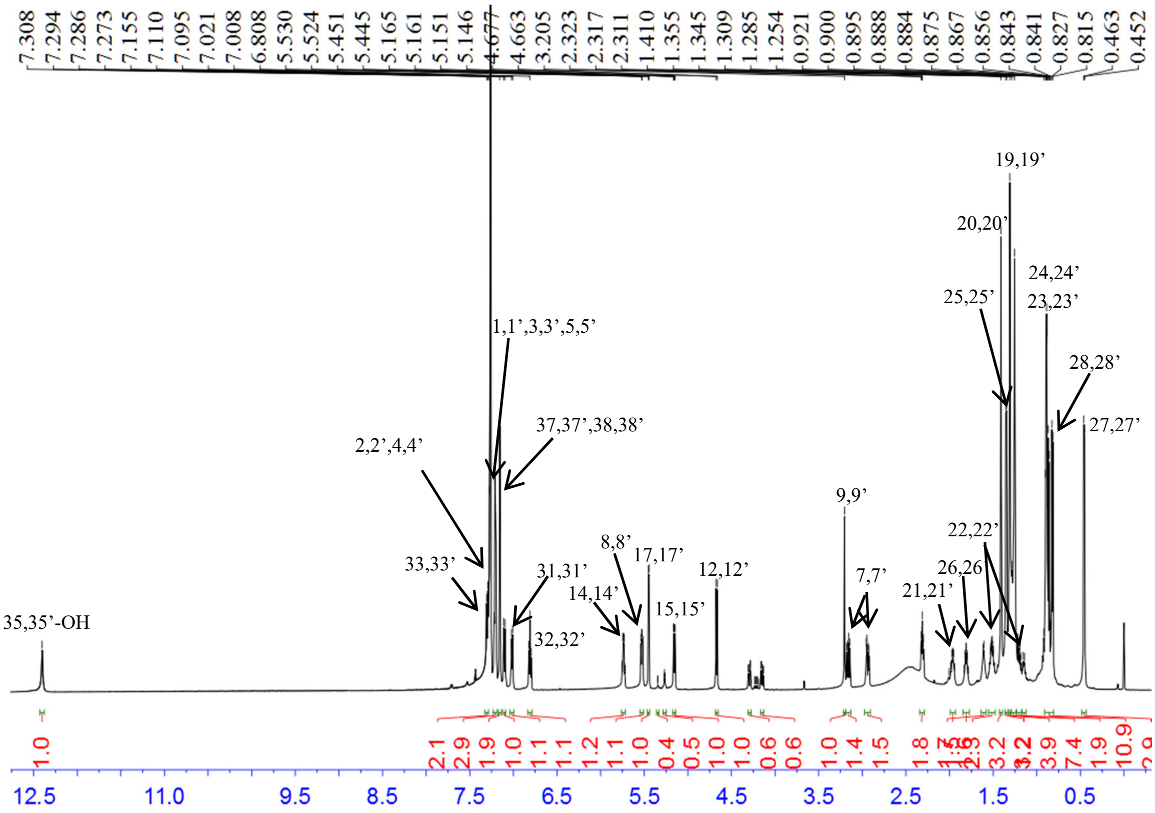

Figure S2. ^{13}C NMR spectrum of **1** in $CDCl_3$ (150 MHz)

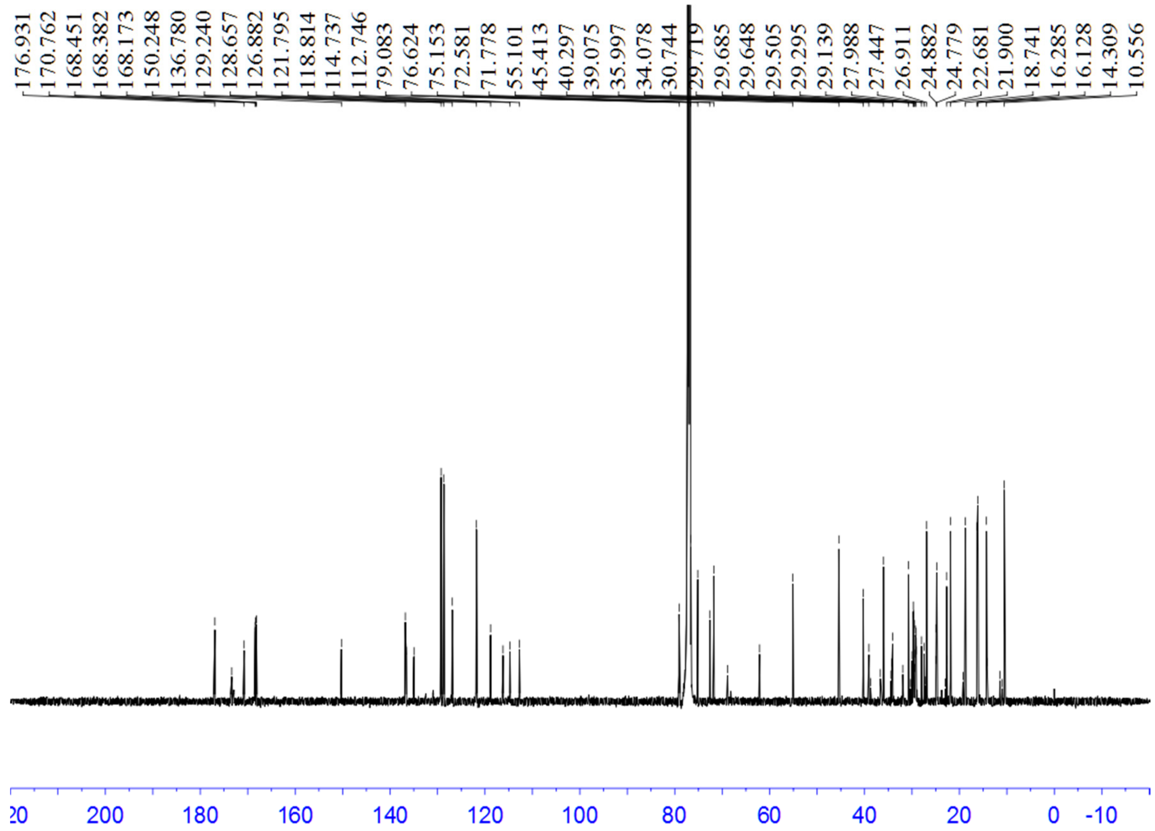

Figure S3. DEPT135 NMR spectrum of **1** in $CDCl_3$ (600 MHz)

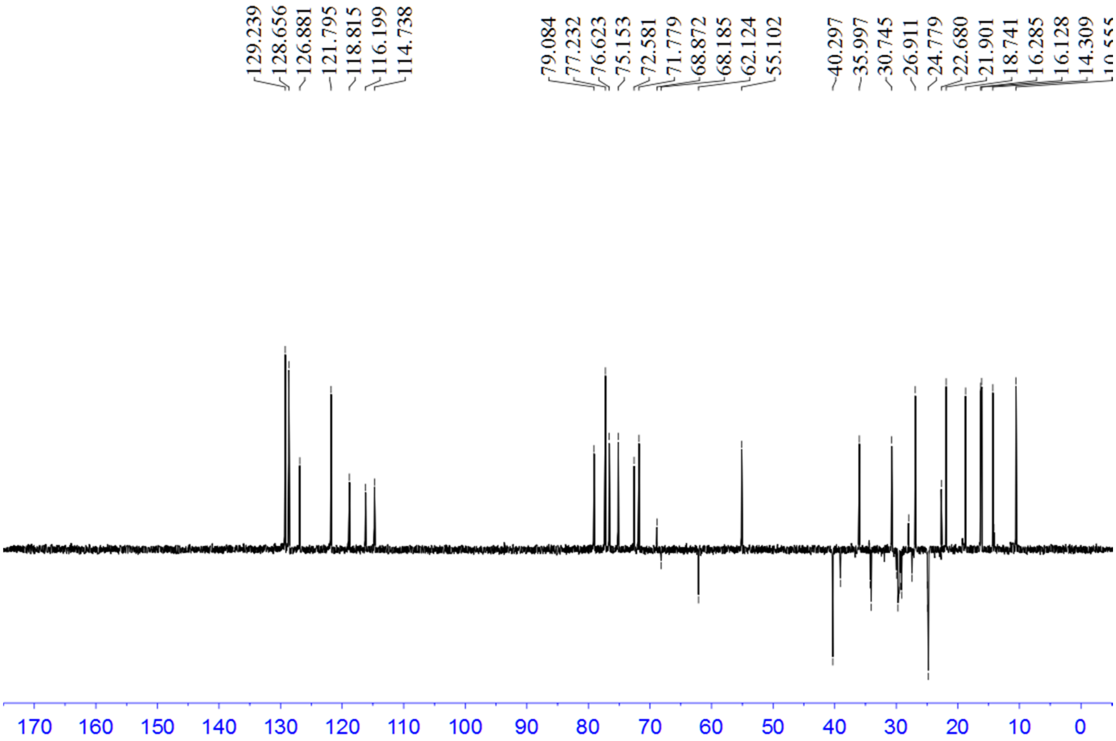

Figure S4. ^1H-^1H COSY NMR spectrum of **1** in $CDCl_3$ (600 MHz)

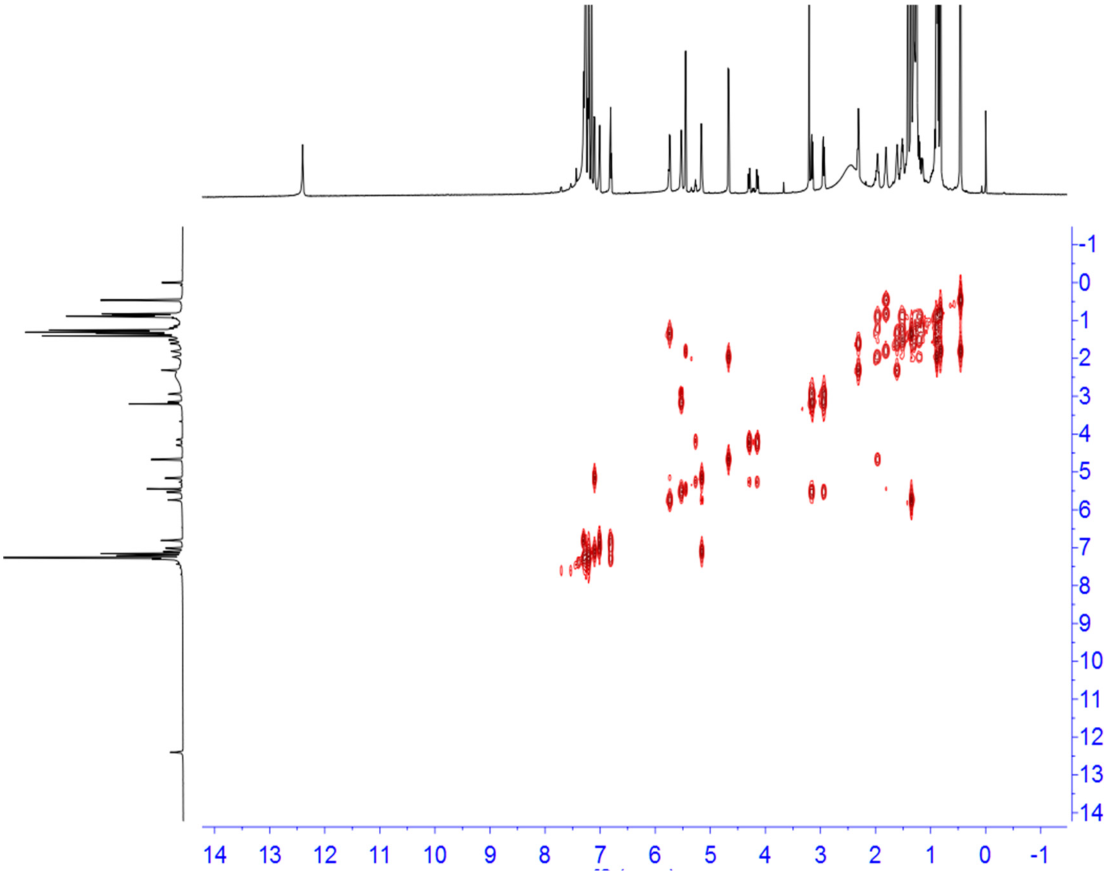

Figure S5. HSQC NMR spectrum of **1** in $CDCl_3$ (600 MHz)

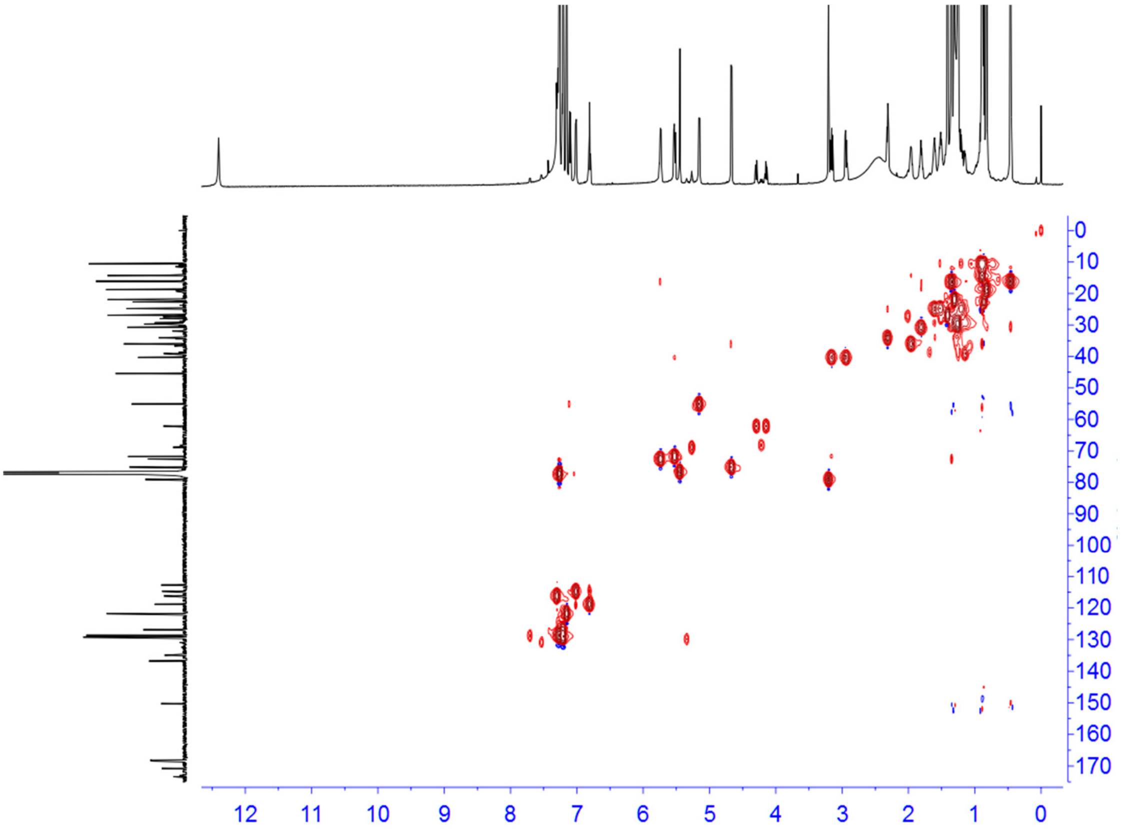

Figure S6. HMBC NMR spectrum of **1** in $CDCl_3$ (600 MHz)

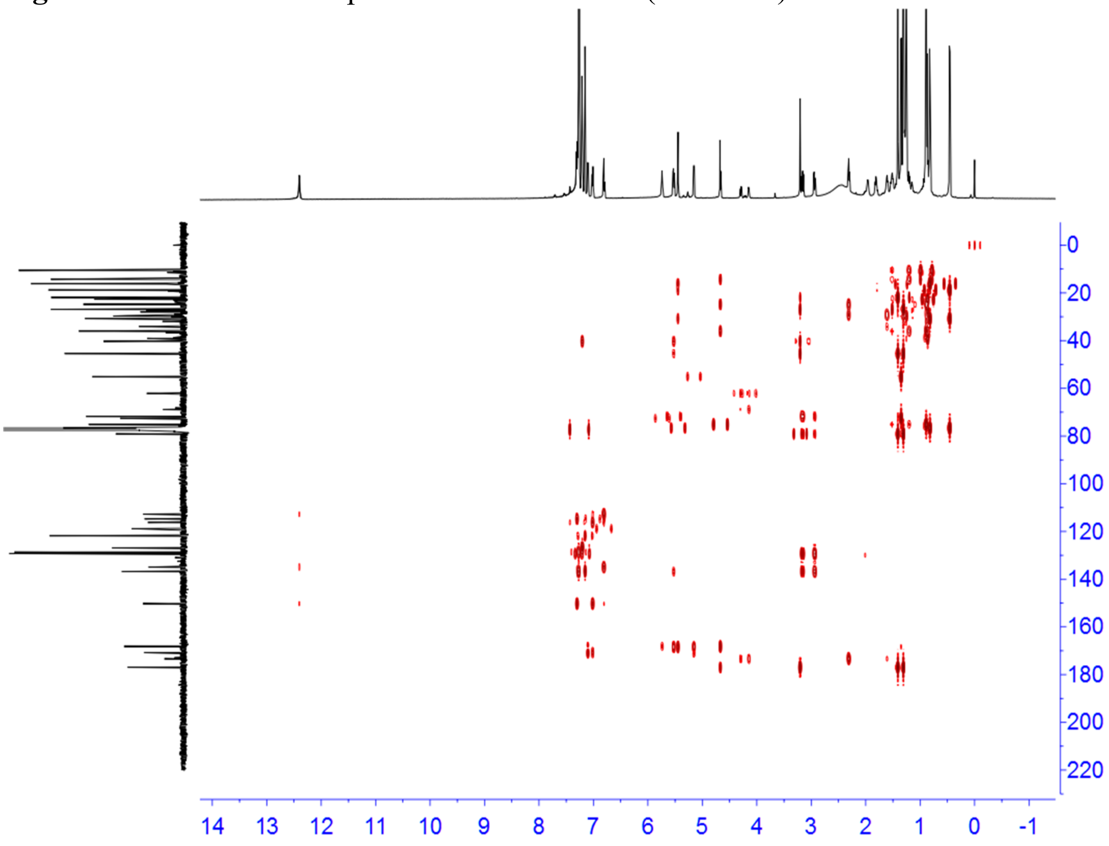

Figure S7. NOESY NMR spectrum of **1** in $CDCl_3$ (600 MHz)

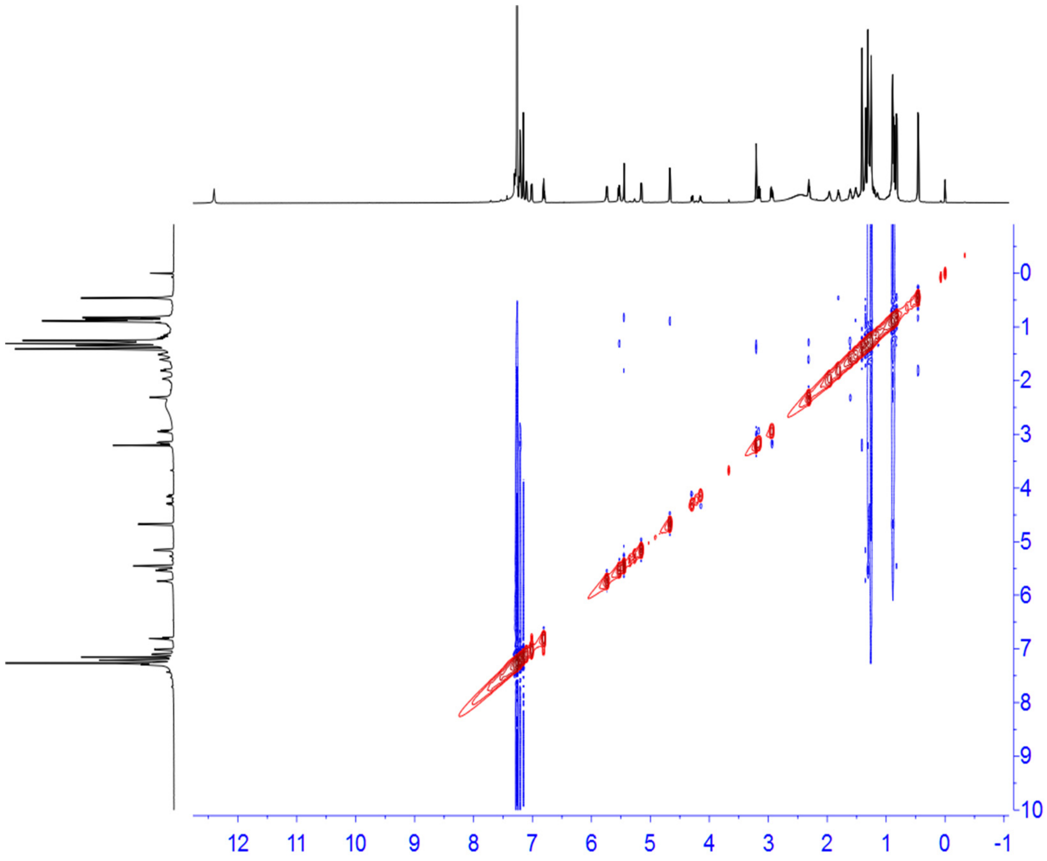

Figure S8. H-N HMBC NMR spectrum of **1** in $CDCl_3$ (600 MHz)

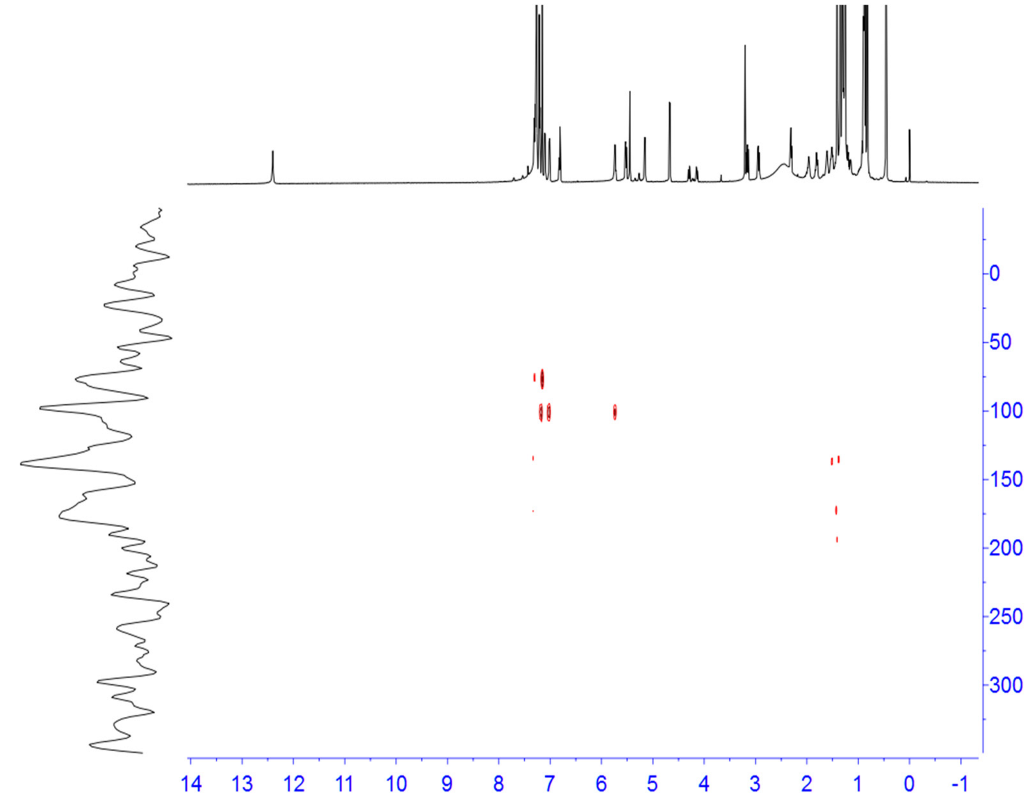

Figure S9. ^{1}H NMR spectrum of **2** in $CDCl_3$ (400 MHz)

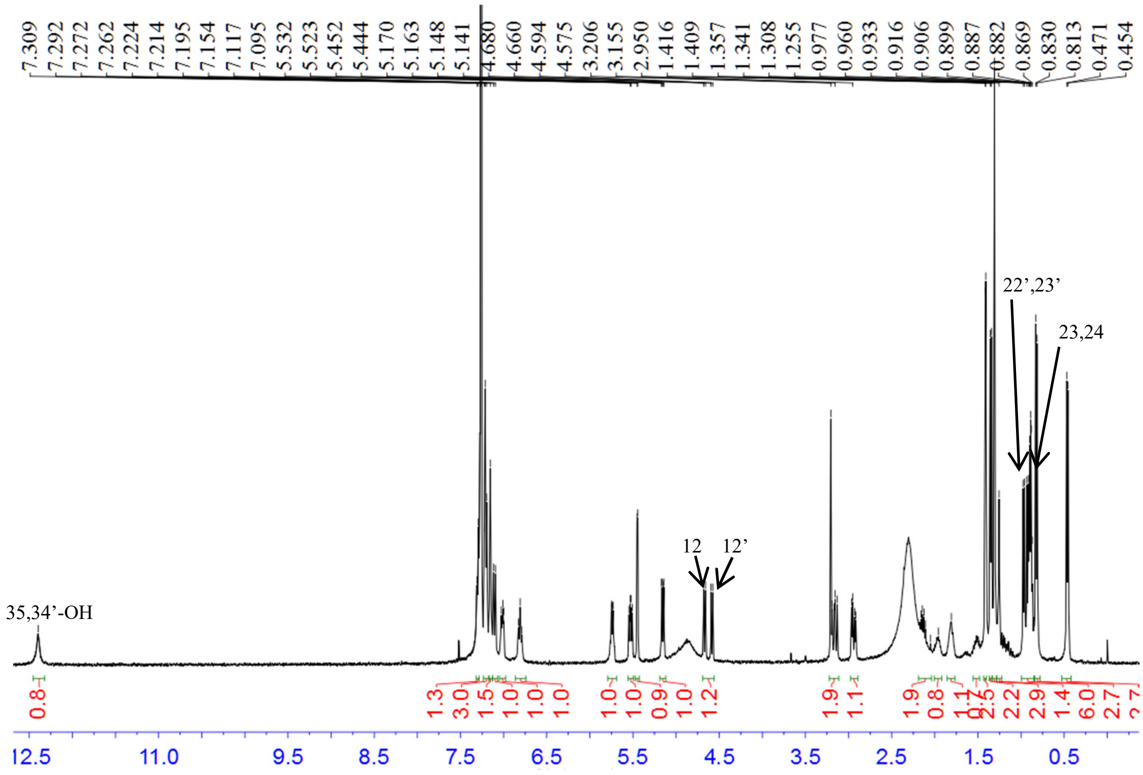

Figure S10. ^{13}C NMR spectrum of **2** in $CDCl_3$ (100 MHz)

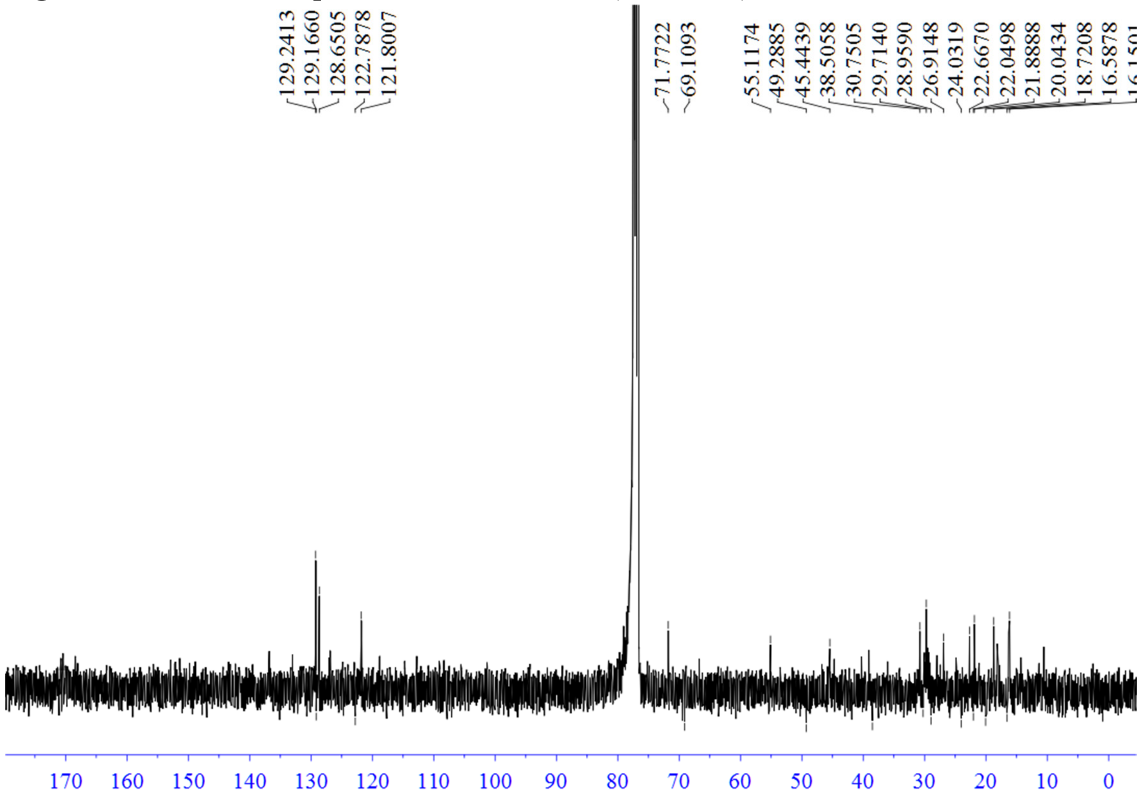

Figure S11. DEPT135 NMR spectrum of **2** in $CDCl_3$ (400 MHz)

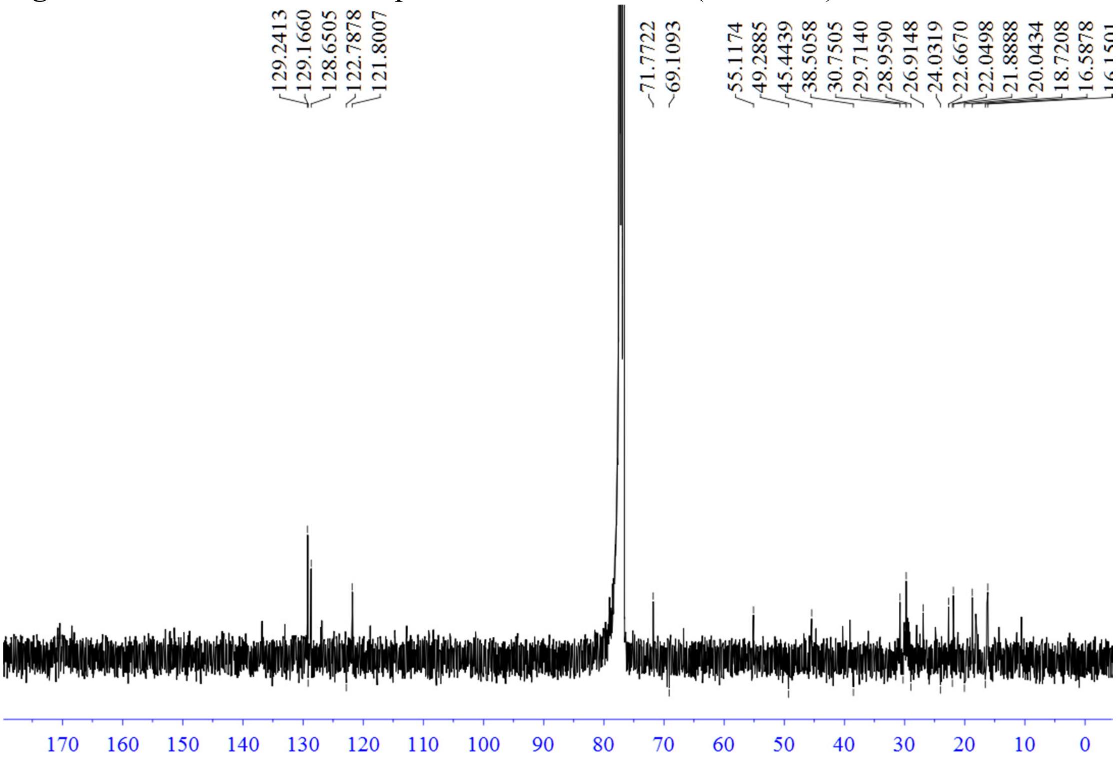

Figure S12. ^{1}H-^{1}H COSY NMR spectrum of **2** in $CDCl_3$ (400 MHz)

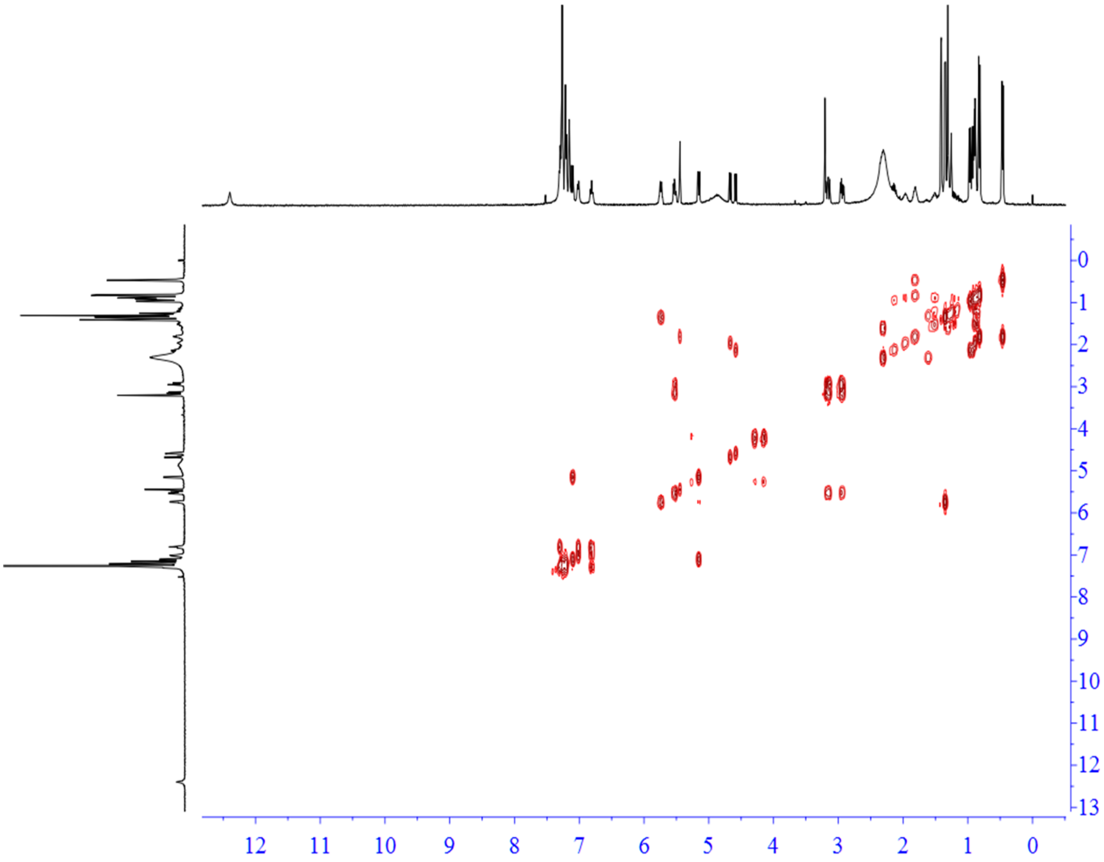

Figure S13. HSQC NMR spectrum of **2** in $CDCl_3$ (400 MHz)

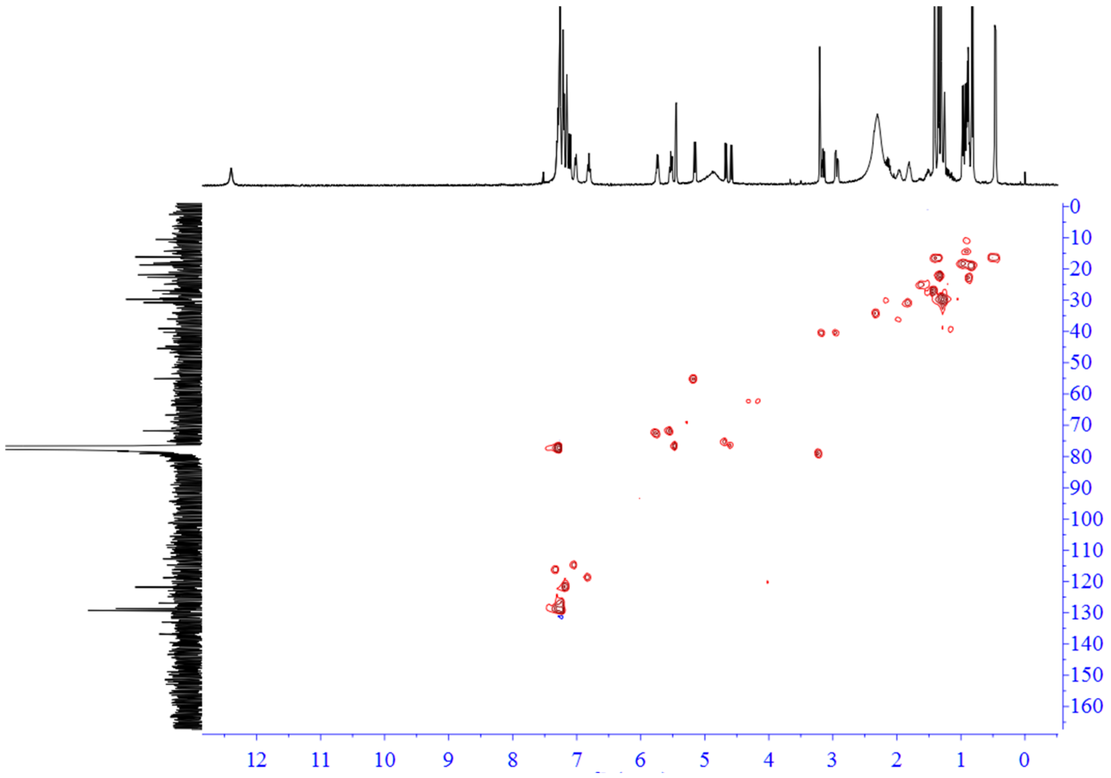

Figure S14. HMBC NMR spectrum of **2** in $CDCl_3$ (400 MHz)

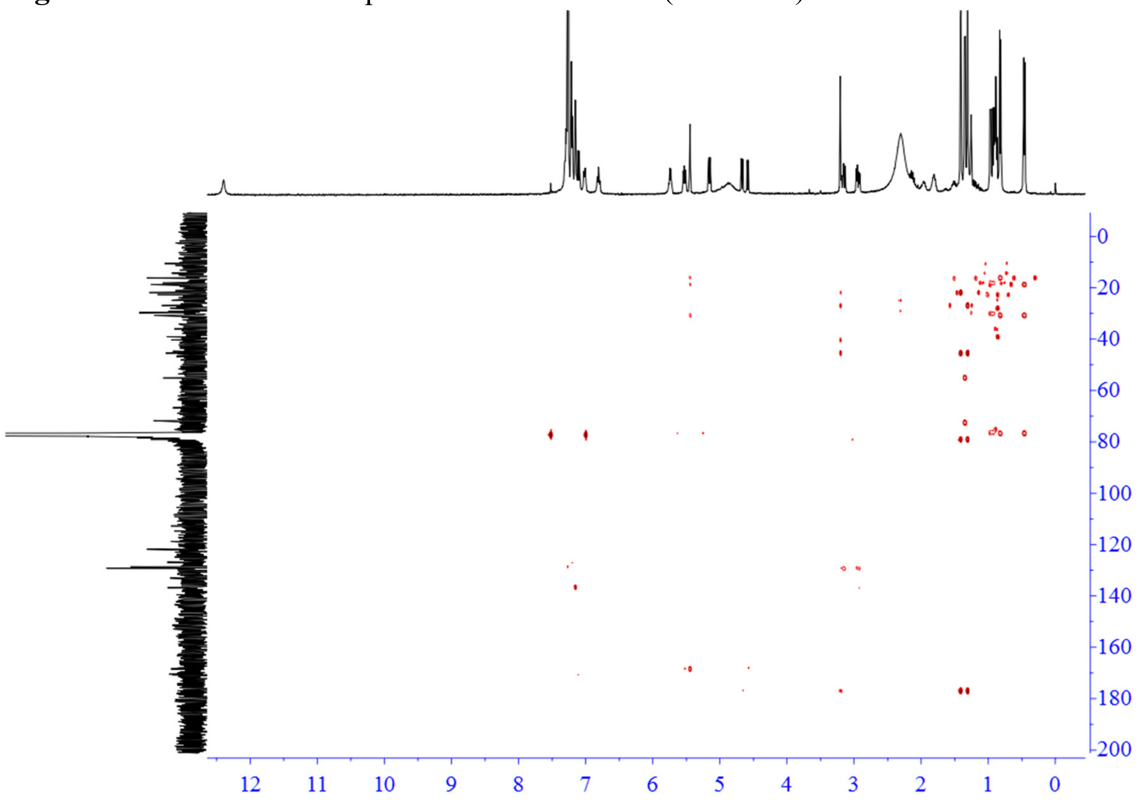

Figure S15. NOESY NMR spectrum of **2** in $CDCl_3$ (400 MHz)

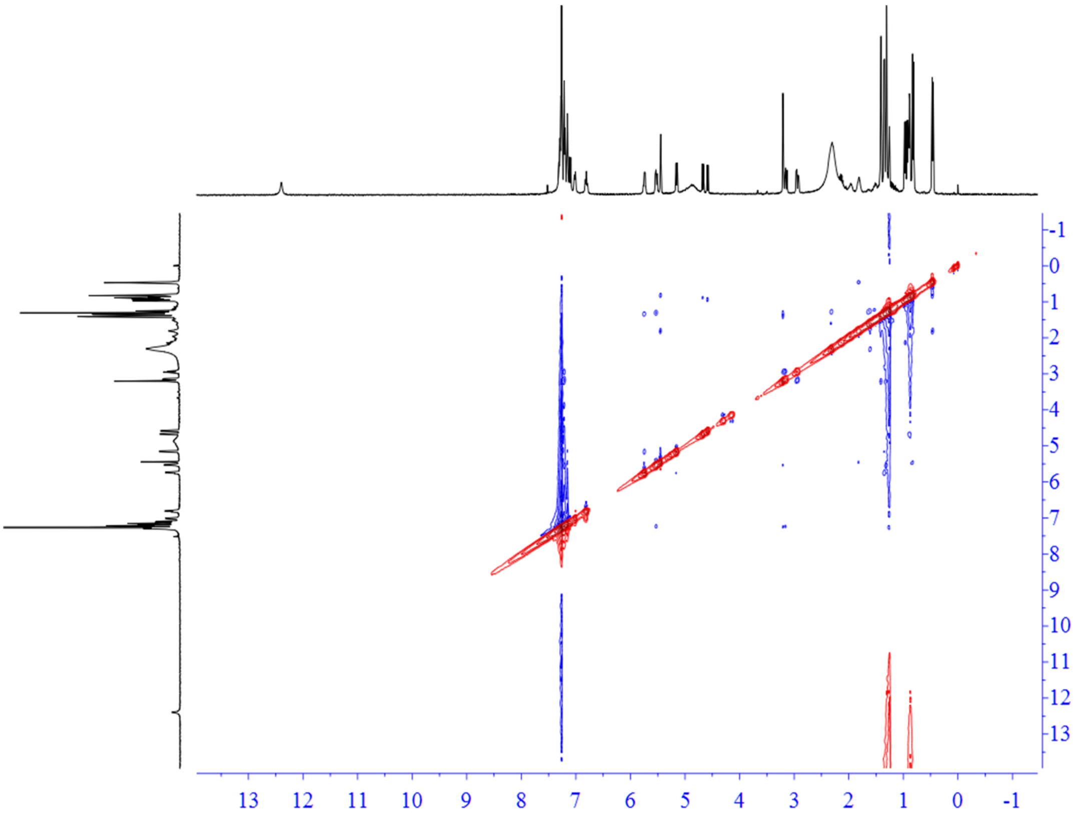

Figure S16. ^{1}H NMR spectrum of **3** in $CDCl_3$ (400 MHz)

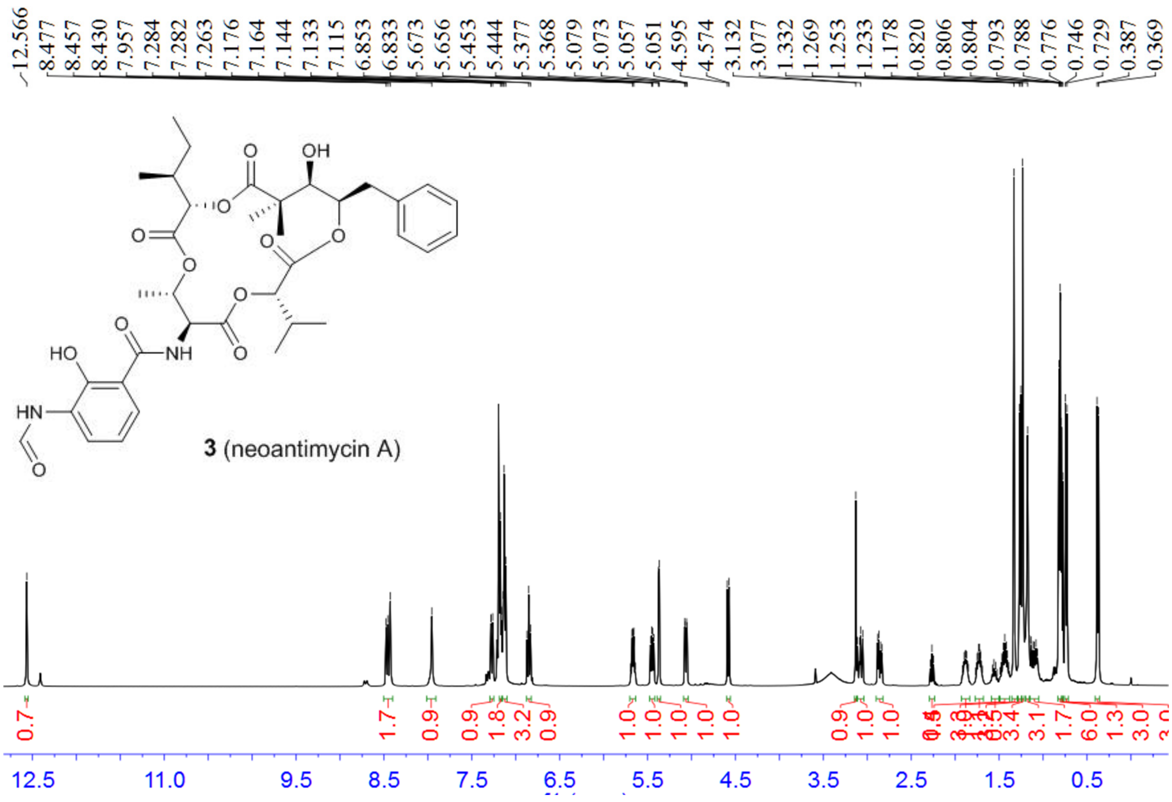

Figure S17. ^{1}H NMR spectrum of **4** in $CDCl_3$ (400 MHz)

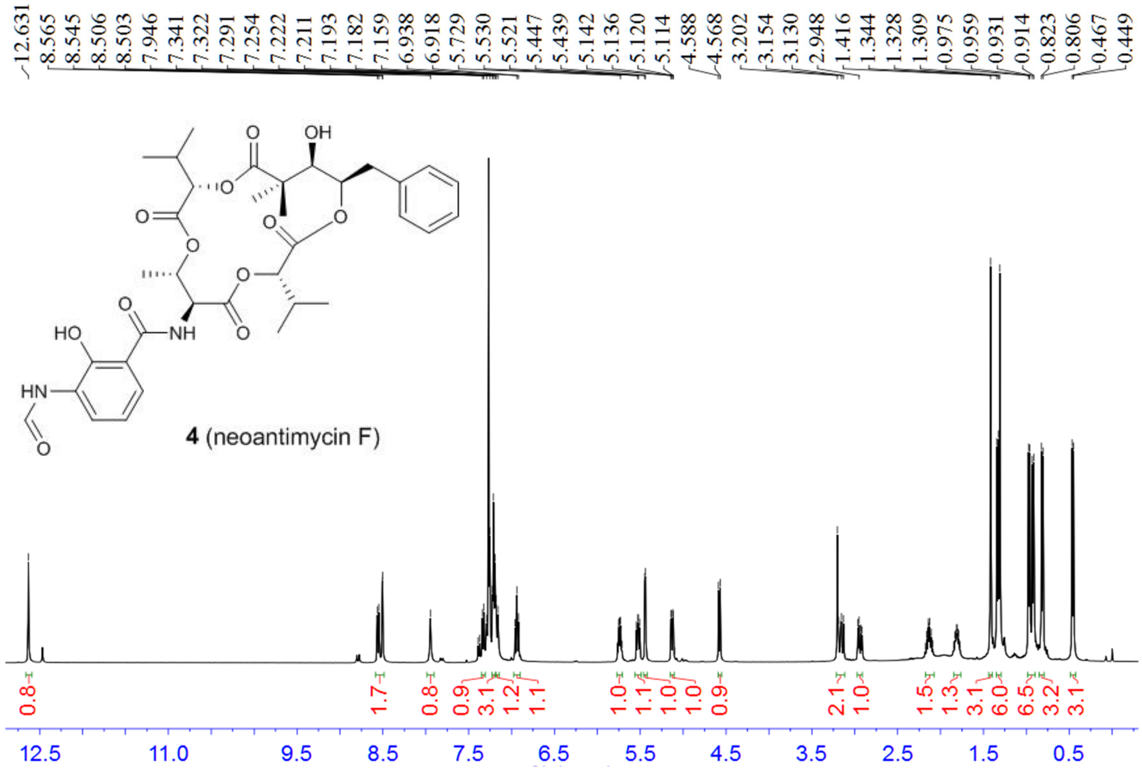

Figure S18. ^{1}H NMR spectrum of **5** in $CDCl_3$ (400 MHz)

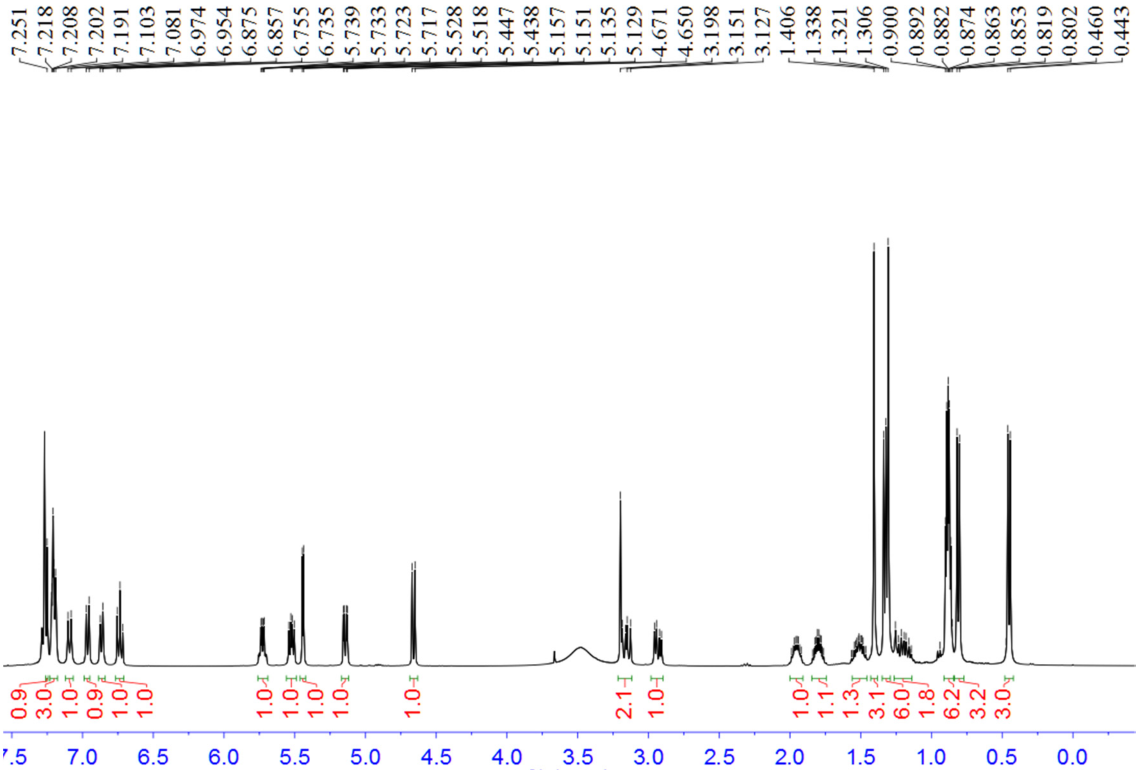

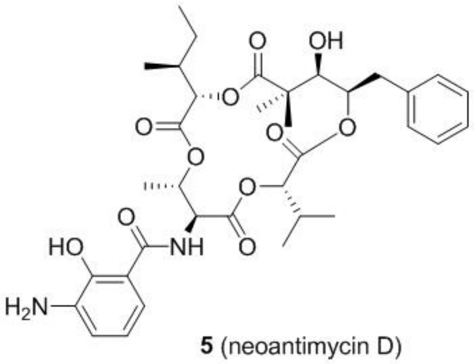

5 (neoantimycin D)

Figure S19. ^{1}H NMR spectrum of **6** in $CDCl_3$ (400 MHz)

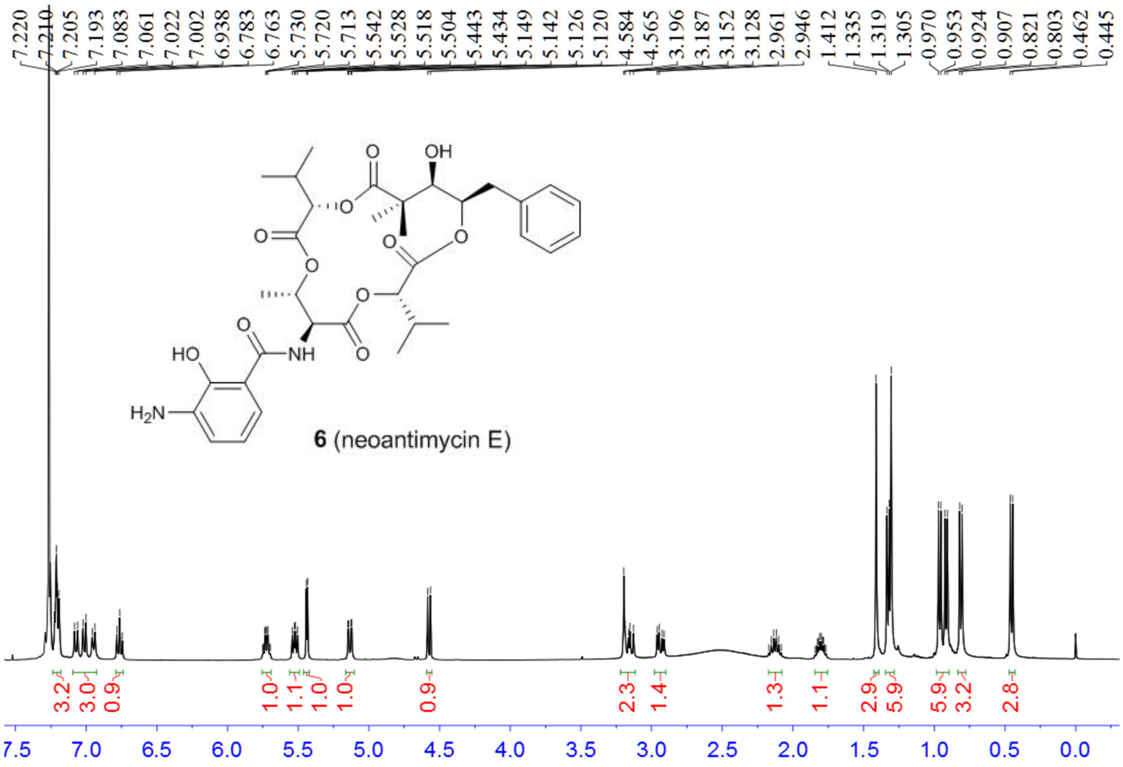

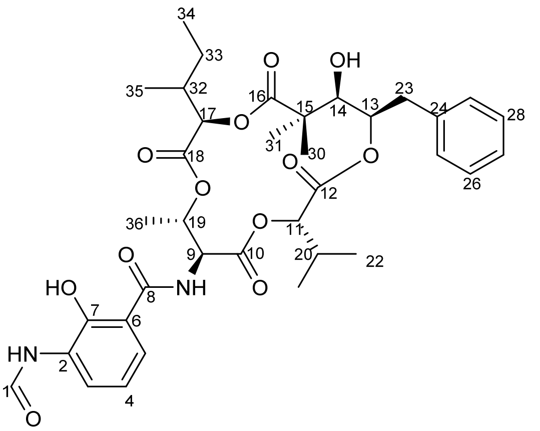

Figure S20. The structure of compound **3**

Table S1. NMR data for compound **3** in $CDCl_3$ (400 MHz)

position	δ_C	δ_H (mult., J)	position	δ_C	δ_H (mult., J)
1	159.3, CH	8.43, s	22	18.8, CH_3	0.74, d (6.9)
2	127.4, C		23a	40.4, CH_2	2.86, dd (14.0,9.6)
3	120.5,CH	7.28, dd (8.0,1.0)	23b		3.08, dd (14.0,9.6)
4	119.0,CH	6.85, t (8.0)	24	136.9, C	
5	124.9,CH	8.47, d (8.0)	25	128.7, CH	7.22, m
6	113.1, C		26	129.3, CH	7.23, m
7	150.7, C		27	127.0, CH	7.13,m
8	170.3,C		28	129.3, CH	7.23, m
9	55.3, CH	5.07, dd (8.8,2.5)	29	128.7, CH	7.22, m
10	168.4, C		30	22.0, CH_3	1.23, s
11	76.8, CH	5.37, d (3.5)	31	27.0, CH_3	1.33, s
12	168.4, C		32	36.1, CH	1.88, m
13	71.9, CH	5.45, dd (9.6,5.8)	33a	24.9, CH_2	1.11, m
14	79.1, CH	3.13, s	33b		0.82, m
15	45.5, C		34	10.6 CH_3	0.80, m
16	177.0, C		35	14.0 CH_3	0.81, m
17	75.2, CH	4.58, d (8.3)	36	16.4, CH_3	1.26, d (6.5)
18	168.2, C		NH-1		7.96, s
19	72.5, CH	5.67, qd (6.5,2.5)	OH-7		12.57, s
20	30.8, CH	1.73, m	NH-8	18.8, CH_3	7.17, m
21	16.2, CH_3	0.38, d (6.9)			

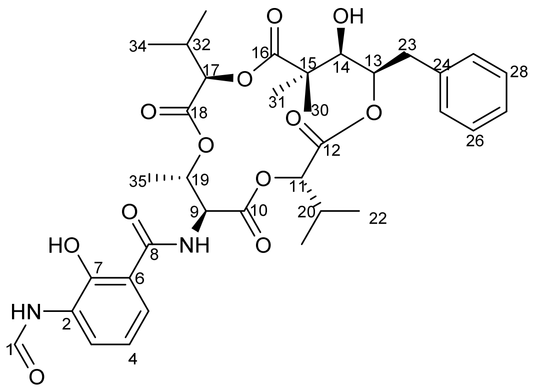

Figure S21. The structure of compound **4**

Table S2. ^{1}H NMR data for compound **4** in $CDCl_3$ (400 MHz)

position	δ_H (mult., J)	position	δ_H (mult., J)
1	8.50, d (1.2)	25	7.22, m
3	7.33, dd (8.0, 1.0)	26	7.23, m
4	6.94, t (8.0)	27	7.13,m
5	8.55, d (8.0)	28	7.23, m
9	5.13, dd (8.8, 2.5)	29	7.22, m
11	5.44, d (3.5)	30	1.31, s
13	5.53, dd (9.6, 5.8)	31	1.42, s
14	3.20, s	32	2.13, m
17	4.58, d (7.8)	33	0.92, d (6.8)
19	5.74, qd (6.5, 2.5)	34	0.97, d (6.8)
20	1.80, m	35	1.34, d (6.5)
21	0.46, d (6.9)	NH-1	7.95, s
22	0.81, d (6.9)	OH-7	12.63, s
23a	2.94, dd (14.0, 9.6)	NH-8	7.25, m
23b	3.16, dd (14.0, 9.6)		

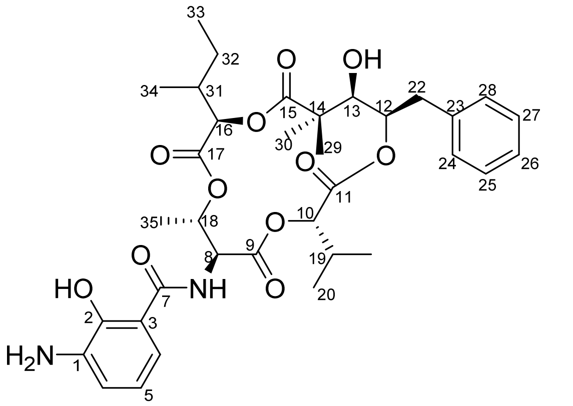

Figure S22. The structure of compound **5**

Table S3. NMR data for compound **5** in $CDCl_3$ (400 MHz)

position	δ_C	δ_H (mult., J)	position	δ_C	δ_H (mult., J)
1	136.8, C		20	16.2, CH_3	0.46, d (5.9)
2	149.8, C		21	18.8, CH_3	0.81, d (6.9)
3	113.0, C		22a	40.4, CH_2	2.93, dd (14.0, 9.6)
4	114.8, CH	6.96, d (7.8)	22b		3.16, dd (14.0, 9.6)
5	119.0,CH	6.74, t (7.8)	23	136.9, C	
6	118.7,CH	6.87, d (7.8)	24	128.7, CH	7.22, m
7	170.8,C		25	129.3, CH	7.23, m
8	55.1, CH	5.14, dd (8.9, 2.6)	26	126.9, CH	7.21,m
9	168.4, C		27	129.3, CH	7.23, m
10	76.7, CH	5.44, d (3.5)	28	128.7, CH	7.22, m
11	168.5, C		29	22.0, CH_3	1.31, s
12	71.8, CH	5.52, dd (9.6, 5.8)	30	27.0, CH_3	1.41, s
13	79.1, CH	3.20, s	31	36.1, CH	1.95, m
14	45.5, C		32a	24.9, CH_2	1.51, m
15	177.0, C		32b		1.19, m
16	75.2, CH	4.66, d (8.3)	33	10.6 CH_3	0.88, m
17	168.2, C		34	14.4, CH_3	0.88, m
18	72.7, CH	5.73, dd (6.5, 2.6)	35	16.3, CH_3	1.33, d (6.5)
19	30.8, CH	1.80, m	NH		7.09, d (8.8)

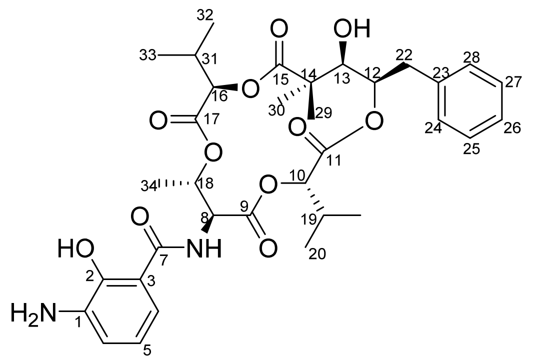

Figure S23. The structure of compound **6**

Table S4. ^{1}H NMR data for compound **6** in $CDCl_3$ (400 MHz)

position	δ_H (mult., J)	position	δ_H (mult., J)
4	7.01, d (8.0)	22b	3.16, dd (14.0, 9.6)
5	6.76, t (8.0)	24	7.22, m
6	6.95, d (8.0)	25	7.23, m
8	5.13, dd (8.9, 2.5)	26	7.21,m
10	5.44, d (3.5)	27	7.23, m
12	5.52, dd (9.6, 5.8)	28	7.22, m
13	3.19, s	29	1.31, s
16	4.57, d (7.9)	30	1.41, s
18	5.73, qd (6.5, 2.5)	31	2.12, m
19	1.80, m	32	0.92, d (6.8)
20	0.46, d (6.9)	33	0.96, d (6.8)
21	0.81, d (6.9)	34	1.33, d (6.5)
22a	2.94, dd (14.0, 9.6)	NH	7.07, d (8.9)

Figure S24. MS spectrum of **1**

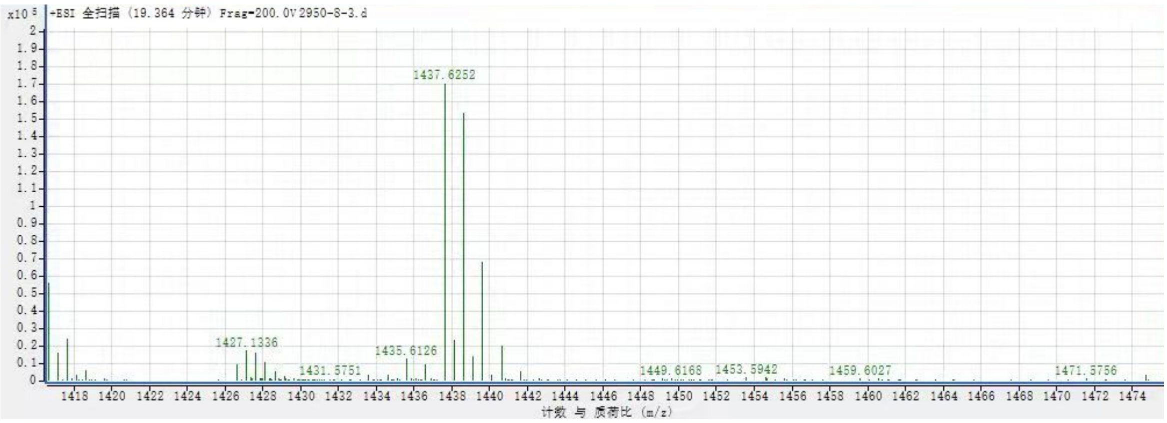

Figure S25. MS spectrum of **2**

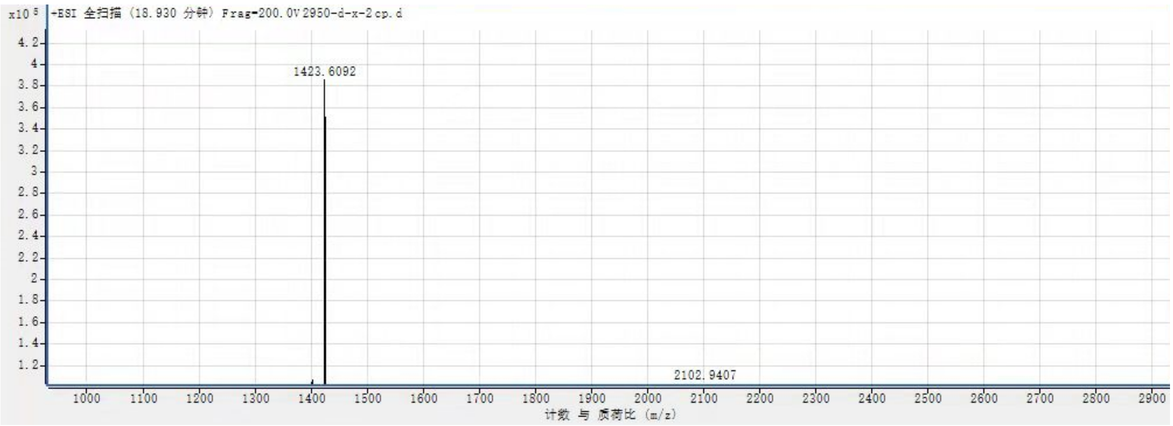

Supplement: Supplementary file 1 [file marinedrugs-19-00624-s001.zip › marinedrugs-1440105-supplementary.pdf]
